# (3*S*)-2-Benzyl-3-carb­oxy-1,2,3,4-tetra­hydro­isoquinolinium chloride monohydrate

**DOI:** 10.1107/S1600536810053122

**Published:** 2010-12-24

**Authors:** Tricia Naicker, Thavendran Govender, Hendrik G. Kruger, Glenn E. M. Maguire

**Affiliations:** a School of Pharmacy and Pharmacology, University of KwaZulu Natal, Durban 4000, South Africa; b School of Chemistry, University of KwaZulu Natal, Durban 4000, South Africa

## Abstract

In the title compound, C_17_H_18_NO_2_^+^·Cl^−^·H_2_O, a precursor to novel asymmetric catalysts, the N-containing six-membered ring of the tetra­hydro­quinolinium unit assumes a half-boat conformation. In the crystal, inter­molecular O—H⋯O, O—H⋯Cl, N—H⋯Cl and C—H⋯O hydrogen bonds and C—H⋯π inter­actions link the mol­ecules into a three-dimensional network.

## Related literature

For related structures of tetra­hydro­isoquinoline derivatives, see: Naicker, Petzold *et al.* (2010 ▶); Naicker, Govender *et al.* (2010 ▶, 2011 ▶); Peters *et al.* (2010 ▶). For related structures with the same chiral centre and conformation of the six-membered ring, see: Naicker *et al.* (2009 ▶); Chakka *et al.* (2010 ▶).
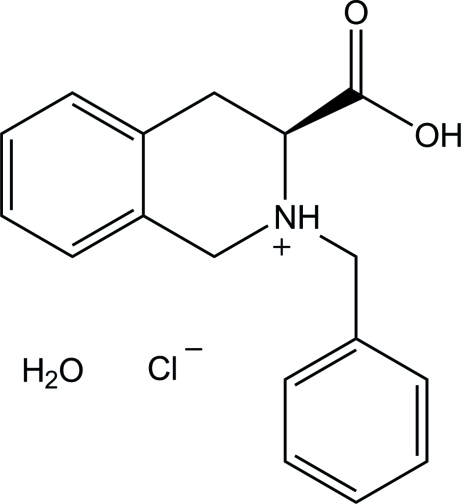


## Experimental

### 

#### Crystal data

C_17_H_18_NO_2_^+^·Cl^−^·H_2_O
*M* *_r_* = 321.79
Monoclinic, 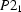

*a* = 8.6159 (8) Å
*b* = 10.0670 (9) Å
*c* = 10.1392 (9) Å
β = 108.686 (2)°
*V* = 833.08 (13) Å^3^
*Z* = 2
Mo *K*α radiation
μ = 0.24 mm^−1^
*T* = 193 K
0.30 × 0.11 × 0.02 mm

#### Data collection

Bruker Kappa DUO APEXII diffractometer
Absorption correction: multi-scan (*SADABS*; Sheldrick, 2008*a* ▶) *T*_min_ = 0.931, *T*_max_ = 0.995
9083 measured reflections
4158 independent reflections
3414 reflections with *I* > 2σ(*I*)
*R*_int_ = 0.024

#### Refinement

*R*[*F* ^2^ > 2σ(*F* ^2^)] = 0.036
*wR*(*F* ^2^) = 0.082
*S* = 1.04
4158 reflections
213 parameters
5 restraints
H atoms treated by a mixture of independent and constrained refinement
Δρ_max_ = 0.19 e Å^−3^
Δρ_min_ = −0.16 e Å^−3^
Absolute structure: Flack (1983 ▶), 1961 Friedel pairs
Flack parameter: −0.01 (5)

### 

Data collection: *APEX2* (Bruker, 2006 ▶); cell refinement: *SAINT* (Bruker, 2006 ▶); data reduction: *SAINT*; program(s) used to solve structure: *SHELXS97* (Sheldrick, 2008*b* ▶); program(s) used to refine structure: *SHELXL97* (Sheldrick, 2008*b* ▶); molecular graphics: *OLEX2* (Dolomanov *et al.*, 2009 ▶); software used to prepare material for publication: *SHELXL97*.

## Supplementary Material

Crystal structure: contains datablocks I, global. DOI: 10.1107/S1600536810053122/is2635sup1.cif

Structure factors: contains datablocks I. DOI: 10.1107/S1600536810053122/is2635Isup2.hkl

Additional supplementary materials:  crystallographic information; 3D view; checkCIF report

## Figures and Tables

**Table 1:** Hydrogen-bond geometry (Å, °) *Cg* is the centroid of the C12–C17 ring.

*D*—H⋯*A*	*D*—H	H⋯*A*	*D*⋯*A*	*D*—H⋯*A*
N1—H1⋯Cl1^i^	0.97 (2)	2.09 (2)	3.0521 (15)	176 (1)
O2—H2⋯O3^ii^	0.96 (2)	1.59 (2)	2.533 (2)	167 (3)
O3—H3*A*⋯Cl1^i^	0.96 (2)	2.21 (2)	3.1615 (15)	172 (2)
O3—H3*B*⋯Cl1	0.96 (2)	2.20 (2)	3.1434 (16)	165 (2)
C9—H9⋯O1^iii^	1.00	2.30	3.169 (2)	145
C15—H15⋯*Cg*^iii^	0.95	2.78	3.386 (3)	122

Symmetry codes: (i) 
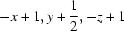
; (ii) 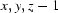; (iii) 
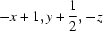
.

**Fig. 1.**
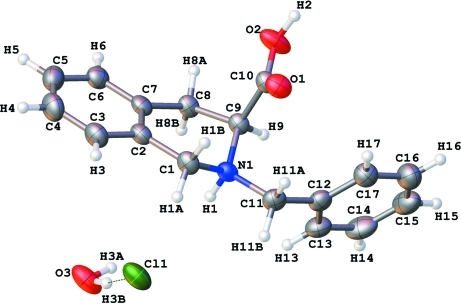
The molecular structure of the title compound with the atom numbering scheme. Displacement ellipsoids are drawn at the 50% probability level.

**Fig. 2.**
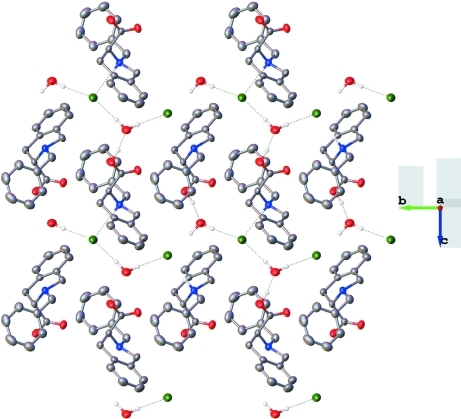
Partial projection viewed along the a axis, depicting hydrogen bonding from the water molecule and chloride ion. Displacement ellipsoids are drawn at the 50% probability level.

## supplementary crystallographic information

### Comment

The tetrahydroisoquinoline (TIQ) molecule and its derivatives have been widely investigated due to their biological and pharmaceutical properties. We have recently had much success with TIQ based ligands for both metal ligand (Peters *et al.*, 2010) and organocatalysis (Naicker, Petzold *et al.*, 2010). Bearing an acid functional group, the title compound is a useful precursor to many of these novel asymmetric catalysts. The neutral form of this compound is commercially available but there has been no report of its single X-ray crystal structure.

The structure has monoclinic (*P*2_1_) symmetry with a single molecule in the asymmetric unit together with a water molecule (Fig. 1). Various intra- and intermolecular short contact interactions (2.87–3.14 Å) occur but only one C15—H···π (C12—C17 ring) is observed within the crystal packing (Table 1). The most significant feature of the structure is the intermolecular hydrogen bonding array. The carboxylic acid functional group (O2—H) hydrogen bonds to the water molecule which in turn interacts with two chloride ions. These ions interact further with another water molecule but also with the protonated tertiary amine nitrogen. This series of interactions helps to construct the three-dimensional network (Fig. 2 and Table 1).

From the crystal structure it is evident that the *N*-containing six membered ring assumes a half boat conformation (Fig. 1), this observation is similar to analogous structures that we have recently reported (Naicker *et al.*, 2009; Naicker, Govender *et al.*, 2010).

### Experimental

(*S*)-Methyl 2-benzyl-1,2,3,4-tetrahydroisoquinoline-3-carboxylate was added to a 10% (v/v) solution of HCl in water (5 mL). The mixture was then microwaved for 2 h at 120 °C, thereafter the reaction mixture was evaporated under reduced pressure to afford the title compound as a white solid.

Melting point 205–208 °C. IR (neat): 3339, 2501, 1712, 1224, 754, 701 cm^-1^. ^1^H NMR (400 MHz, CDCl_3_) δ = 3.28 (d, 1H), 3.36 (d, 1H), 4.37 (m, 5H), 7.13 (d, 1H), 7.25 (m, 3H) and 7.39 (m, 5H).

Recrystallization from 10% HCl in water afforded colourless crystals suitable for X-ray analysis.

### Refinement

All H atoms on carbons were positioned geometrically with C—H distances ranging from 0.95 to 1.00 Å and refined as riding on their parent atoms, with *U*_iso_(H) = 1.2*U*_eq_(C). Atoms H1, H2, H3A and H3B were located in a difference Fourier map. The distances of N1—H1, O2—H2, O3—H3A and O3—H3B were restrained to 0.97 (1) Å and the *U*_iso_ values of H3A and H3B were assigned as 1.2*U*_eq_(O3).

### Crystal data

**Table:**

C_17_H_18_NO_2_^+^·Cl^−^·H_2_O	*F*(000) = 340
*M**_r_* = 321.79	*D*_x_ = 1.283 Mg m^−^^3^
Monoclinic, *P*2_1_	Melting point: 479 K
Hall symbol: P 2yb	Mo *K*α radiation, λ = 0.71073 Å
*a* = 8.6159 (8) Å	Cell parameters from 9083 reflections
*b* = 10.0670 (9) Å	θ = 2.1–28.3°
*c* = 10.1392 (9) Å	µ = 0.24 mm^−^^1^
β = 108.686 (2)°	*T* = 193 K
*V* = 833.08 (13) Å^3^	Needle, colourless
*Z* = 2	0.30 × 0.11 × 0.02 mm

### Data collection

**Table:**

Bruker Kappa DUO APEXII diffractometer	4158 independent reflections
Radiation source: fine-focus sealed tube	3414 reflections with *I* > 2σ(*I*)
graphite	*R*_int_ = 0.024
φ and ω scans	θ_max_ = 28.3°, θ_min_ = 2.1°
Absorption correction: multi-scan (*SADABS*; Sheldrick, 2008a	*h* = −11→11
*T*_min_ = 0.931, *T*_max_ = 0.995	*k* = −13→13
9083 measured reflections	*l* = −13→13

### Refinement

**Table:**

Refinement on *F*^2^	Secondary atom site location: difference Fourier map
Least-squares matrix: full	Hydrogen site location: inferred from neighbouring sites
*R*[*F*^2^ > 2σ(*F*^2^)] = 0.036	H atoms treated by a mixture of independent and constrained refinement
*wR*(*F*^2^) = 0.082	*w* = 1/[σ^2^(*F*_o_^2^) + (0.0365*P*)^2^ + 0.0675*P*] where *P* = (*F*_o_^2^ + 2*F*_c_^2^)/3
*S* = 1.04	(Δ/σ)_max_ < 0.001
4158 reflections	Δρ_max_ = 0.19 e Å^−^^3^
213 parameters	Δρ_min_ = −0.16 e Å^−^^3^
5 restraints	Absolute structure: Flack (1983), 1961 Friedel pairs
Primary atom site location: structure-invariant direct methods	Flack parameter: −0.01 (5)

### Special details

**Table:**

Experimental. Half sphere of data collected using the Bruker *SAINT* software package. Crystal to detector distance = 30 mm; combination of φ and ω scans of 0.5°, 30 s per °, 2 iterations
Geometry. All e.s.d.'s (except the e.s.d. in the dihedral angle between two l.s. planes) are estimated using the full covariance matrix. The cell e.s.d.'s are taken into account individually in the estimation of e.s.d.'s in distances, angles and torsion angles; correlations between e.s.d.'s in cell parameters are only used when they are defined by crystal symmetry. An approximate (isotropic) treatment of cell e.s.d.'s is used for estimating e.s.d.'s involving l.s. planes.
Refinement. Refinement of *F*^2^ against ALL reflections. The weighted *R*-factor *wR* and goodness of fit *S* are based on *F*^2^, conventional *R*-factors *R* are based on *F*, with *F* set to zero for negative *F*^2^. The threshold expression of *F*^2^ > σ(*F*^2^) is used only for calculating *R*-factors(gt) *etc*. and is not relevant to the choice of reflections for refinement. *R*-factors based on *F*^2^ are statistically about twice as large as those based on *F*, and *R*- factors based on ALL data will be even larger.

### Fractional atomic coordinates and isotropic or equivalent isotropic displacement parameters (Å^2^)

**Table:**

	*x*	*y*	*z*	*U*_iso_*/*U*_eq_	
Cl1	0.49392 (7)	0.38386 (5)	0.55522 (5)	0.04878 (14)	
O1	0.52160 (16)	0.58413 (12)	−0.04364 (13)	0.0387 (3)	
O2	0.6338 (2)	0.76556 (14)	−0.10233 (14)	0.0490 (4)	
H2	0.629 (4)	0.716 (3)	−0.184 (2)	0.090 (9)*	
O3	0.6423 (2)	0.66136 (15)	0.67265 (14)	0.0555 (4)	
H3A	0.610 (3)	0.7278 (19)	0.601 (2)	0.067*	
H3B	0.578 (3)	0.5841 (17)	0.634 (2)	0.067*	
N1	0.49861 (17)	0.70659 (13)	0.19862 (14)	0.0280 (3)	
H1	0.501 (2)	0.7664 (16)	0.2736 (15)	0.038 (5)*	
C1	0.6027 (2)	0.59202 (17)	0.27205 (18)	0.0327 (4)	
H1A	0.5619	0.5600	0.3472	0.039*	
H1B	0.5925	0.5182	0.2052	0.039*	
C2	0.7801 (2)	0.62971 (17)	0.33336 (17)	0.0310 (4)	
C3	0.8823 (2)	0.5464 (2)	0.43363 (19)	0.0409 (5)	
H3	0.8377	0.4710	0.4650	0.049*	
C4	1.0477 (3)	0.5725 (2)	0.4878 (2)	0.0483 (5)	
H4	1.1170	0.5144	0.5550	0.058*	
C5	1.1130 (2)	0.6837 (2)	0.4441 (2)	0.0441 (5)	
H5	1.2268	0.7026	0.4819	0.053*	
C6	1.0113 (2)	0.7668 (2)	0.34518 (18)	0.0368 (4)	
H6	1.0564	0.8426	0.3149	0.044*	
C7	0.8446 (2)	0.74187 (16)	0.28922 (17)	0.0302 (4)	
C8	0.7346 (2)	0.83776 (17)	0.18686 (17)	0.0305 (4)	
H8A	0.7920	0.8686	0.1221	0.037*	
H8B	0.7157	0.9162	0.2385	0.037*	
C9	0.5689 (2)	0.77988 (16)	0.10185 (16)	0.0271 (3)	
H9	0.4946	0.8568	0.0638	0.033*	
C10	0.5722 (2)	0.69626 (16)	−0.02157 (17)	0.0292 (3)	
C11	0.3223 (2)	0.66112 (18)	0.1368 (2)	0.0354 (4)	
H11A	0.3204	0.5756	0.0880	0.043*	
H11B	0.2765	0.6452	0.2133	0.043*	
C12	0.2151 (2)	0.75869 (18)	0.03655 (19)	0.0343 (4)	
C13	0.1818 (2)	0.8829 (2)	0.0808 (2)	0.0411 (4)	
H13	0.2281	0.9074	0.1759	0.049*	
C14	0.0813 (3)	0.9713 (2)	−0.0132 (3)	0.0522 (6)	
H14	0.0603	1.0566	0.0173	0.063*	
C15	0.0118 (3)	0.9354 (3)	−0.1511 (3)	0.0551 (6)	
H15	−0.0581	0.9958	−0.2150	0.066*	
C16	0.0432 (3)	0.8130 (2)	−0.1963 (2)	0.0512 (5)	
H16	−0.0053	0.7886	−0.2911	0.061*	
C17	0.1460 (2)	0.7249 (2)	−0.1032 (2)	0.0417 (4)	
H17	0.1694	0.6408	−0.1351	0.050*	

### Atomic displacement parameters (Å^2^)

**Table:**

	*U*^11^	*U*^22^	*U*^33^	*U*^12^	*U*^13^	*U*^23^
Cl1	0.0783 (4)	0.0361 (2)	0.0339 (2)	−0.0070 (3)	0.0209 (2)	0.0013 (2)
O1	0.0530 (8)	0.0247 (6)	0.0402 (7)	−0.0058 (6)	0.0177 (6)	−0.0053 (6)
O2	0.0828 (11)	0.0376 (8)	0.0353 (7)	−0.0184 (7)	0.0311 (7)	−0.0053 (6)
O3	0.0928 (12)	0.0438 (9)	0.0322 (7)	−0.0064 (8)	0.0231 (8)	−0.0007 (7)
N1	0.0354 (7)	0.0231 (7)	0.0269 (7)	−0.0014 (6)	0.0120 (6)	0.0022 (6)
C1	0.0427 (10)	0.0236 (8)	0.0313 (9)	−0.0004 (7)	0.0111 (7)	0.0065 (7)
C2	0.0415 (10)	0.0274 (8)	0.0249 (8)	0.0019 (7)	0.0116 (7)	0.0006 (7)
C3	0.0514 (12)	0.0373 (10)	0.0329 (10)	0.0028 (9)	0.0121 (9)	0.0067 (8)
C4	0.0530 (13)	0.0483 (12)	0.0370 (10)	0.0094 (10)	0.0050 (9)	0.0085 (10)
C5	0.0384 (10)	0.0502 (12)	0.0391 (10)	0.0028 (9)	0.0061 (8)	−0.0071 (9)
C6	0.0408 (10)	0.0375 (10)	0.0324 (9)	−0.0053 (8)	0.0119 (8)	−0.0057 (8)
C7	0.0404 (9)	0.0276 (9)	0.0231 (8)	0.0001 (7)	0.0111 (7)	−0.0032 (7)
C8	0.0379 (9)	0.0232 (8)	0.0293 (8)	−0.0051 (7)	0.0092 (7)	−0.0001 (7)
C9	0.0351 (9)	0.0181 (7)	0.0281 (8)	−0.0011 (7)	0.0100 (7)	0.0030 (7)
C10	0.0367 (9)	0.0223 (8)	0.0274 (8)	−0.0004 (7)	0.0086 (7)	0.0015 (7)
C11	0.0369 (10)	0.0309 (9)	0.0406 (10)	−0.0051 (8)	0.0154 (8)	0.0026 (8)
C12	0.0312 (9)	0.0319 (9)	0.0422 (9)	−0.0031 (7)	0.0150 (7)	0.0035 (8)
C13	0.0399 (10)	0.0397 (10)	0.0492 (10)	0.0035 (9)	0.0220 (8)	−0.0013 (11)
C14	0.0511 (13)	0.0396 (11)	0.0771 (16)	0.0143 (10)	0.0360 (12)	0.0076 (11)
C15	0.0482 (13)	0.0631 (15)	0.0600 (14)	0.0194 (11)	0.0256 (11)	0.0236 (12)
C16	0.0491 (12)	0.0586 (14)	0.0448 (12)	0.0056 (10)	0.0135 (10)	0.0106 (11)
C17	0.0430 (11)	0.0393 (11)	0.0428 (10)	−0.0021 (9)	0.0138 (9)	−0.0004 (9)

### Geometric parameters (Å, °)

**Table:**

O1—C10	1.205 (2)	C6—H6	0.9500
O2—C10	1.310 (2)	C7—C8	1.509 (2)
O2—H2	0.961 (10)	C8—C9	1.527 (2)
O3—H3A	0.957 (10)	C8—H8A	0.9900
O3—H3B	0.963 (10)	C8—H8B	0.9900
N1—C9	1.502 (2)	C9—C10	1.516 (2)
N1—C1	1.504 (2)	C9—H9	1.0000
N1—C11	1.516 (2)	C11—C12	1.500 (3)
N1—H1	0.965 (9)	C11—H11A	0.9900
C1—C2	1.502 (3)	C11—H11B	0.9900
C1—H1A	0.9900	C12—C13	1.390 (3)
C1—H1B	0.9900	C12—C17	1.392 (3)
C2—C3	1.392 (2)	C13—C14	1.386 (3)
C2—C7	1.394 (2)	C13—H13	0.9500
C3—C4	1.379 (3)	C14—C15	1.382 (3)
C3—H3	0.9500	C14—H14	0.9500
C4—C5	1.388 (3)	C15—C16	1.371 (3)
C4—H4	0.9500	C15—H15	0.9500
C5—C6	1.382 (3)	C16—C17	1.388 (3)
C5—H5	0.9500	C16—H16	0.9500
C6—C7	1.388 (2)	C17—H17	0.9500
			
C10—O2—H2	110.4 (19)	C9—C8—H8B	108.7
H3A—O3—H3B	106 (2)	H8A—C8—H8B	107.6
C9—N1—C1	113.50 (13)	N1—C9—C10	112.63 (13)
C9—N1—C11	116.00 (13)	N1—C9—C8	108.58 (13)
C1—N1—C11	109.44 (13)	C10—C9—C8	114.61 (14)
C9—N1—H1	107.3 (12)	N1—C9—H9	106.9
C1—N1—H1	103.3 (12)	C10—C9—H9	106.9
C11—N1—H1	106.2 (11)	C8—C9—H9	106.9
C2—C1—N1	112.18 (13)	O1—C10—O2	125.28 (17)
C2—C1—H1A	109.2	O1—C10—C9	124.90 (16)
N1—C1—H1A	109.2	O2—C10—C9	109.79 (14)
C2—C1—H1B	109.2	C12—C11—N1	113.61 (14)
N1—C1—H1B	109.2	C12—C11—H11A	108.8
H1A—C1—H1B	107.9	N1—C11—H11A	108.8
C3—C2—C7	119.88 (17)	C12—C11—H11B	108.8
C3—C2—C1	118.13 (15)	N1—C11—H11B	108.8
C7—C2—C1	121.95 (15)	H11A—C11—H11B	107.7
C4—C3—C2	120.48 (18)	C13—C12—C17	118.93 (18)
C4—C3—H3	119.8	C13—C12—C11	121.11 (17)
C2—C3—H3	119.8	C17—C12—C11	119.95 (17)
C3—C4—C5	119.98 (18)	C14—C13—C12	120.24 (19)
C3—C4—H4	120.0	C14—C13—H13	119.9
C5—C4—H4	120.0	C12—C13—H13	119.9
C6—C5—C4	119.49 (18)	C15—C14—C13	120.1 (2)
C6—C5—H5	120.3	C15—C14—H14	120.0
C4—C5—H5	120.3	C13—C14—H14	120.0
C5—C6—C7	121.30 (18)	C16—C15—C14	120.3 (2)
C5—C6—H6	119.4	C16—C15—H15	119.8
C7—C6—H6	119.4	C14—C15—H15	119.8
C6—C7—C2	118.85 (16)	C15—C16—C17	119.9 (2)
C6—C7—C8	120.32 (15)	C15—C16—H16	120.0
C2—C7—C8	120.78 (16)	C17—C16—H16	120.0
C7—C8—C9	114.30 (14)	C16—C17—C12	120.5 (2)
C7—C8—H8A	108.7	C16—C17—H17	119.8
C9—C8—H8A	108.7	C12—C17—H17	119.8
C7—C8—H8B	108.7		
			
C9—N1—C1—C2	47.47 (18)	C11—N1—C9—C8	170.53 (14)
C11—N1—C1—C2	178.80 (14)	C7—C8—C9—N1	46.10 (19)
N1—C1—C2—C3	163.47 (15)	C7—C8—C9—C10	−80.80 (18)
N1—C1—C2—C7	−18.8 (2)	N1—C9—C10—O1	1.5 (2)
C7—C2—C3—C4	−1.3 (3)	C8—C9—C10—O1	126.28 (19)
C1—C2—C3—C4	176.48 (18)	N1—C9—C10—O2	179.67 (14)
C2—C3—C4—C5	1.1 (3)	C8—C9—C10—O2	−55.54 (19)
C3—C4—C5—C6	−0.7 (3)	C9—N1—C11—C12	−37.9 (2)
C4—C5—C6—C7	0.5 (3)	C1—N1—C11—C12	−167.86 (14)
C5—C6—C7—C2	−0.7 (3)	N1—C11—C12—C13	−65.2 (2)
C5—C6—C7—C8	176.86 (17)	N1—C11—C12—C17	115.34 (18)
C3—C2—C7—C6	1.1 (2)	C17—C12—C13—C14	0.0 (3)
C1—C2—C7—C6	−176.63 (16)	C11—C12—C13—C14	−179.48 (18)
C3—C2—C7—C8	−176.46 (16)	C12—C13—C14—C15	1.0 (3)
C1—C2—C7—C8	5.9 (2)	C13—C14—C15—C16	−0.8 (3)
C6—C7—C8—C9	162.44 (15)	C14—C15—C16—C17	−0.3 (3)
C2—C7—C8—C9	−20.1 (2)	C15—C16—C17—C12	1.3 (3)
C1—N1—C9—C10	66.57 (17)	C13—C12—C17—C16	−1.1 (3)
C11—N1—C9—C10	−61.44 (18)	C11—C12—C17—C16	178.37 (18)
C1—N1—C9—C8	−61.46 (17)		

### Hydrogen-bond geometry (Å, °)

**Table:**

Cg is the centroid of the C12–C17 ring.

**Table:**

*D*—H···*A*	*D*—H	H···*A*	*D*···*A*	*D*—H···*A*
N1—H1···Cl1^i^	0.97 (2)	2.09 (2)	3.0521 (15)	176.(1)
O2—H2···O3^ii^	0.96 (2)	1.59 (2)	2.533 (2)	167 (3)
O3—H3A···Cl1^i^	0.96 (2)	2.21 (2)	3.1615 (15)	172 (2)
O3—H3B···Cl1	0.96 (2)	2.20 (2)	3.1434 (16)	165 (2)
C9—H9···O1^iii^	1.00	2.30	3.169 (2)	145
C15—H15···Cg^iii^	0.95	2.78	3.386 (3)	122

Symmetry codes: (i) −*x*+1, *y*+1/2, −*z*+1; (ii) *x*, *y*, *z*−1; (iii) −*x*+1, *y*+1/2, −*z*.
